# Inhibition of the Anti-Apoptotic Bcl-2 Family by BH3 Mimetics Sensitize the Mitochondrial Permeability Transition Pore Through Bax and Bak

**DOI:** 10.3389/fcell.2021.765973

**Published:** 2021-12-01

**Authors:** Pooja Patel, Arielys Mendoza, Dexter J. Robichaux, Meng C. Wang, Xander H. T. Wehrens, Jason Karch

**Affiliations:** ^1^ Department of Molecular Physiology and Biophysics, Baylor College of Medicine, Houston, TX, United States; ^2^ Cardiovascular Research Institute, Baylor College of Medicine, Houston, TX, United States; ^3^ Huffington Center on Aging, Department of Molecular and Human Genetics, Baylor College of Medicine, Houston, TX, United States; ^4^ Department of Molecular and Human Genetics, Baylor College of Medicine, Houston, TX, United States; ^5^ Howard Hughes Medical Institute, Baylor College of Medicine, Houston, TX, United States

**Keywords:** mitochondria, permeability transition, BCL-2 family, BH3 mimetics, necrosis, mitochondrial dysfunction, calcium

## Abstract

Mitochondrial permeability transition pore (MPTP)-dependent necrosis contributes to numerous pathologies in the heart, brain, and skeletal muscle. The MPTP is a non-selective pore in the inner mitochondrial membrane that is triggered by high levels of matrix Ca^2+^, and sustained opening leads to mitochondrial dysfunction. Although the MPTP is defined by an increase in inner mitochondrial membrane permeability, the expression of pro-apoptotic Bcl-2 family members, Bax and Bak localization to the outer mitochondrial membrane is required for MPTP-dependent mitochondrial dysfunction and subsequent necrotic cell death. Contrary to the role of Bax and Bak in apoptosis, which is dependent on their oligomerization, MPTP-dependent necrosis does not require oligomerization as monomeric/inactive forms of Bax and Bak can facilitate mitochondrial dysfunction. However, the relationship between Bax and Bak activation/oligomerization and MPTP sensitization remains to be explored. Here, we use a combination of *in vitro* and *ex vivo* approaches to determine the role of the anti-apoptotic Bcl-2 family members, which regulate Bax/Bak activity, in necrotic cell death and MPTP sensitivity. To study the role of each predominantly expressed anti-apoptotic Bcl-2 family member (i.e., Mcl-1, Bcl-2, and Bcl-xL) in MPTP regulation, we utilize various BH3 mimetics that specifically bind to and inhibit each. We determined that the inhibition of each anti-apoptotic Bcl-2 family member lowers mitochondrial calcium retention capacity and sensitizes MPTP opening. Furthermore, the inhibition of each Bcl-2 family member exacerbates both apoptotic and necrotic cell death *in vitro* in a Bax/Bak-dependent manner. Our findings suggests that mitochondrial Ca^2+^ retention capacity and MPTP sensitivity is influenced by Bax/Bak activation/oligomerization on the outer mitochondrial membrane, providing further evidence of the crosstalk between the apoptotic and necrotic cell death pathways.

## Introduction

The Bcl-2 family members are critical regulators of apoptotic cell death by regulating mitochondrial outer membrane permeabilization (MOMP) through the activation or inhibition of Bax and Bak oligomerization (Westphal et al., 2014; Kale et al., 2018). During MOMP the outer mitochondrial membrane is permeable to proteins up to 100 kDa in size (Kalkavan and Green, 2018). Notably, this event leads to the release of cytochrome-c, which promotes apoptosome formation and subsequent caspase activation resulting in apoptotic cell death (Kalkavan and Green, 2018). Conversely, mitochondrial permeability transition pore (MPTP)-dependent necrosis is a pathological form of regulated cell death that occurs when high levels of matrix Ca^2+^ leads to MPTP opening (Karch and Molkentin, 2015). The MPTP resides on the inner mitochondrial membrane and is permeable to solutes up to 1.5 kDa when open (Karch and Molkentin, 2015). Sustained MPTP opening leads to mitochondrial membrane potential collapse, mitochondrial swelling, and dysfunction, which typically results in necrotic cell death (Kwong and Molkentin, 2015). Previous research has identified two key regulators of the MPTP: the adenine nucleotide translocase (ANT) family and cyclophilin D (CypD). The ANT family is a group of proteins that exchange ADP and ATP across the inner mitochondrial membrane (Woodfield et al., 1998). Whereas, CypD is a cis-trans peptidyl-prolyl isomerase that resides in the mitochondrial matrix (Halestrap et al., 1997). Genetic deletion and pharmacological inhibition of either of these regulators leads to a desensitization of MPTP and increases mitochondrial Ca^2+^ retention capacity (Halestrap and Brenner, 2003; Kokoszka et al., 2004; Baines et al., 2005; Nakagawa et al., 2005).

Previously, the MOMP and MPTP were viewed as distinct entities (Karch et al., 2015). However, recent work has implicated that both permeabilization events involve Bax and Bak (Karch et al., 2013). Indeed, deletion of both Bax/Bak renders cells resistant to both apoptotic and necrotic cell death (Karch et al., 2013). However, differing from apoptosis, Bax/Bak activation and oligomerization is not required for MPTP-dependent necrosis and MPTP-dependent mitochondrial dysfunction (Karch et al., 2013). Although the inactive/monomeric forms of Bax and Bak are sufficient for necrotic cell death, activated/oligomeric Bax/Bak may further sensitize MPTP opening and necrotic cell death (Karch et al., 2013; Karch et al., 2015; Patel and Karch, 2020). The other pro-apoptotic (Bcl-2 homology (BH) 3 only members) or anti-apoptotic (BH1-4 domain containing member) Bcl-2 family members regulate Bax/Bak activation (Tsujimoto, 1998). Since anti-apoptotic Bcl-2 family members are often upregulated in many types of cancers, researchers have developed small molecule drugs that bind to the BH3 domain of these members to inhibit their anti-apoptotic properties, thus sensitizing cells to apoptosis by MOMP through Bax and Bak activation (Nakajima and Tanaka, 2016; Villalobos-Ortiz et al., 2020). Previously, mitochondria treated with BH3 mimetics displayed disorganized cristae, suggesting that inhibition of anti-apoptotic Bcl-2 family members play a role in mitochondrial dysfunction (Henz et al., 2019). Here, we investigate how the inhibition of individual and/or multiple anti-apoptotic Bcl-2 family members by BH3 mimetics, ABT-199 (Bcl-2 inhibitor), A-1331852 (Bcl-xL inhibitor), S63845 (Mcl-1 inhibitor), ABT-737 (Bcl-2 and Bcl-xL inhibitor), and Obatoclax (Pan-anti-apoptotic Bcl-2 family inhibitor), affect MPTP sensitivity and necrotic cell death.

## Materials and Methods

### Animal Models

Wild type (WT) C57BL/6J and *Ppif* ^*−/−*^ 2–3 month old male and female mice were utilized for the *ex vivo* mitochondrial isolations. *Ppif* ^*−/−*^ mice were previously generated as described (Baines et al., 2005; Nakagawa et al., 2005). All experimental procedures with animals were approved by the Institutional Animal Care and Use Committee of Baylor College of Medicine, protocols IACUC AN-7915. All mice were treated humanely as per compliance with the rules and regulations of animal care and euthanasia under this committee. The minimal number of mice were used in this study to attain statistical significance using a two-tailed Student T-test. Both male and female mice were.

### MEF Mitochondrial Isolation

WT and *Bax/Bak1* double knockout (DKO) SV40 immortalized mouse embryonic fibroblasts (MEFs) were plated on six 245 mm × 245 mm plates (Corning, 431110) and were cultured until reaching 100% confluency. Plates were washed with 10 ml mitochondrial isolation buffer (225 mM mannitol, 75 mM sucrose, 5 mM HEPES, 1 mM EGTA, pH 7.4). Cells were then scraped in 7 ml isolation buffer per plate and collected into a 50 ml conical tube. The suspension was pelleted at 800 x g for 5 min at 4°C. The supernatant was discarded and the pellet was suspended in 7 ml of isolation buffer before homogenization by a Teflon/glass tissue grinder (5–10 strokes). The homogenate was brought up to 12 ml isolation buffer before centrifugation at 800 *g* for 5 min. Then the supernatant was transferred to a fresh tube and centrifuged again at 800 x g for 5 min. Then the supernatant was transferred to another fresh tube and spun at 10,000 x g for 10 min. The supernatant was aspirated, and the pellet was washed with 7 ml of isolation buffer and was then centrifuged at 10,000 x g for 10 min. All centrigfugation was performed at 4°C and samples were kept on ice. The final pellet was suspended in 1 ml of KCl buffer (125 mM KCl, 20 mM HEPES, 2 mM KH_2_PO_4_, 40 µM EGTA, pH 7.2). Mitochondrial concentration was quantified using a NanoDrop.

### Liver Mitochondrial Isolation

Whole livers were isolated from WT and *Ppif* ^*−/−*^ mice and washed in mitochondrial isolation buffer then minced into 1–2 mm pieces in 7 ml of mitochondrial isolation buffer. The tissue was homogenized using a glass and Teflon/glass tissue grinder (8–10 strokes). All steps were performed on ice. The liver homogenates were then centrifuged at 800 x g for 5 min, and the supernatants were then collected and centrifuged at 10,000 x g for 10 min. The supernatant was aspirated, and the pellet was washed with 7 ml of isolation buffer and was then centrifuged at 10,000 x g for 10 min. All centrifugations were performed at 4°C. The pellet from the last spin was suspended in 1 ml KCL buffer and mitochondrial concentration was measured using a NanoDrop.

### Calcium Retention Capacity and Mitochondrial Swelling Assays

Two mg of isolated mitochondria were suspended in a total volume of 1 ml consisting KCl buffer, 1 mM Malic acid (Sigma-Aldrich), 7 mM Pyruvate (Sigma-Aldrich), and 50 nM Calcium Green 5N (Invitrogen) in a quartz cuvette, which was placed inside the fluorimeter (PTI QuantaMaster 800, Horiba Scientific). Calcium uptake was measured by fluorescent emission of Calcium Green 5N. Simultaneously, mitochondrial swelling was measured by transmittance light. Each BH3 mimetic was used at concentrations: 0.5, 1, 10, 50, 100 nM, and 1 µM. Final BH3 mimetics concentrations used throughout the study were 200 nM ABT-199 (Selleckchem.com, S8048), 100 nM A-1331852 (abcam, ab218112), 100 nM ABT-737 (EMD Millipore Sigma, 197333), 10 nM S63845 (Selleckchem.com, S8383), 10 nM Obatoclax (Selleckchem, GX15-070). For some experiments ADP (300 uM) (Sigma-Aldrich, A2754) and/or CsA (2 uM) (Sigma-Aldrich, 30024) were used to desensitize the MPTP. CaCl_2_ (20 μM, 40 μM, and 80 µM) (Sigma-Adrich, C4901) was added into this system in succession until MPTP opening occurred, indicated by an upturn in Calcium Green 5N fluorescence, or when mitochondria were saturated with Ca^2+^ and were no longer able to take up further additions of CaCl_2_, indicated by a stair-stacking in the Ca^2+^ uptake graphs.

### Tissue Culture and Analysis of Cell Death

WT and *Bax/Bak1* DKO MEFs were cultured in Iscove’s Modified Dulbecco’s Medium (IMDM) (Cytiva, SH30228.01) supplemented with 10% bovine growth serum (HyClone, SH30541.03), 1 mM penicillin streptomycin (gibco, 15140-122), and 1 mM nonessential amino acids (gibco, 11140-050). For cell death experiments, these cells were plated in 12-well plates and were treated with cell death inducing agents when they were 85–90% confluent. The cells were treated with BH3 mimetics ABT-199 (30 µM), A-1331852 (10 µM), ABT-737 (10 µM), S63845 (10 µM), and Obatoclax (500 nM) with and without ionomycin (5 µM for 20 h) or staurosporine (5 nM for 12 h). Cell death was determined by propidium iodide (PI) positivity (BioVision, Milpitas, CA). When quantifying cell death, the media was collected into a microcentrifuge tube and the cells were trypsinized and placed into their appropriate tube of media. The cells were then centrifuged for 5 min at 5,000 x g and the cell pellet was suspended in Hank’s Balanced Salt solution (HBSS) containing 0.01% bovine serum albumin (BSA) and 0.1% PI and incubated for 10 min. The cells were then quantified for PI positivity at 10,000 counts per sample using a Guava® easyCyte 5HT HPL Benchtop Flow Cytometer (Millipore Sigma).

### Western Blotting

All Western blots were performed from MEF and liver mitochondrial lysates (isolated as previously described). Following mitochondrial isolations, the mitochondrial pellets were suspended in radioimmunoprecipitation (RIPA) buffer (10 mM Tris-HCl pH7.49, 100 nM NaCl, 1 mM EDTA, 1 nM EGTA, 1% Triton X-100, 10% glycerol, 0.1% SDS, 0.5% sodium deoxycholate) containing protease inhibitor cocktails (Roche). The samples were then sonicated, and the insoluble fractions were discarded following centrifugation (21,000 x g for 10 min at 4°C). SDS sample buffer (250 mM Tris-HCl pH 7.0, 10% SDS, 5% β-mercaptoethanol, 0.02% bromophenol blue, 30% glycerol) was added to the lysates, and samples were boiled for 5 min at 100°C. The samples were then loaded onto to 12% SDS-PAGE gels and then transferred onto polyvinylidene fluoride transfer membranes (MilliporeSigma). Prior to primary antibody incubation, the membranes were blocked in tris-buffered saline, 0.1% Tween (TBST) containing 4% dry milk. The following primary antibodies were used: Bcl-xL (Cell Signaling Technology; 54H6; 1:2000), Mcl-1 (ROCKLAND, 600-401-394S, 1:500), Bcl-2 (abcam, ab182858,1:500), and Total OXPHOS rodent WB antibody cocktail (that contained antibodies to complexes 1 to 5, ComII, or ComV as labeled in Western blots) (abcam; ab110413; 1:10,000). Following overnight incubation with the primary antibody, the membranes were washed 3 times for 5 min with 1X TBST. These blots were then incubated in their respective secondary antibody, goat-anti-mouse IgG (H + L) Secondary Alkaline phosphatase (NOVUS, NB7536; 1:10,000) or goat anti-rabbit IgG (H + L) Secondary Alkaline phosphatase (NOVUS, NB7157; 1:10,000) for 2 h. Before imaging, the membranes were washed 5 times for 5 min each with 1X TBST. These blots were incubated in ECF substrate for 1 min before imaging using the iBright imaging system (ThermoFisher).

### Mass Spectrometry With Tandem Mass Tags

Anti-apoptotic Bcl-2 family (Bcl-2, Bcl-xL, Mcl-1) protein expression was determined in 5 WT liver and 5 MEF mitochondrial crude fractions using quantitative tandem mass tags (TMT10plex #90110, Pierce) (Parks et al., 2019). Protein extracts from each liver or MEF mitochondrial fraction were reduced, alkylated, then digested overnight before being labeled with TMT Reagents before being mixed and fractionated. These labeled samples were analyzed by high resolution Thermo Scientific^TM^ Orbitrap^TM^ Mass Spectrometry. Each protein amount was quantified as a percent of total anti-apoptotic Bcl-2 protein amount.

### Statistical Analysis

The data is presented as the mean with the error bars representing the standard error of the mean (SEM). When comparing two groups, an unpaired two-tailed Student’s t test was performed and when comparing multiple groups, a one-way ANOVA was performed followed by a Bonferroni Post Hoc analysis for multiple comparisons using GraphPad Prism. All values were considered statistically significant when *p* < 0.05 or *p* < 0.005 as labeled in the figure legends. The sample number of biological replicates for each experiment is indicated in the figure legends.

## Results

### BH3 Mimetics Exacerbate Necrotic Cell Death in Mouse Embryonic Fibroblasts

**FIGURE 1:**
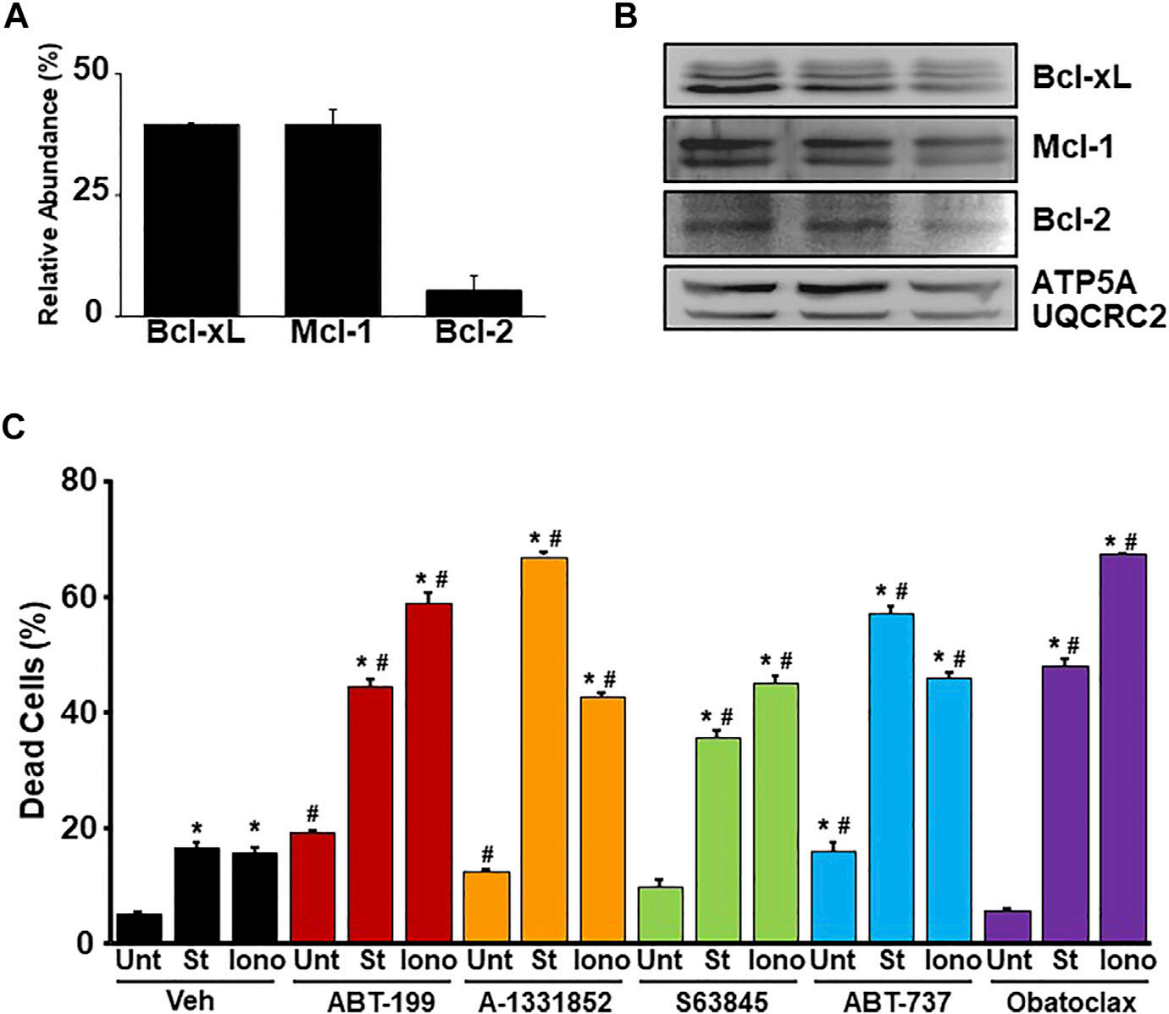
BH3 mimetics sensitize MEFs to apoptosis and necrosis. **(A)** Quantitation of the relative abundance of anti-apoptotic Bcl-2 family members (Bcl-2, Bcl-xL, and Mcl-1) from enriched mitochondrial fractions isolated from WT MEFs measured by tandem-mass-tagging mass spectrometry (n = 5). **(B)** Western blots for anti-apoptotic Bcl-2 family proteins (Bcl-2, Bcl-xL, and Mcl-1) and Ox. Phos. (ATP5A and UQCRC2) (loading control) using enriched mitochondrial lysates isolated from WT MEFs. **(C)** Flow cytometer analysis of cell death measured by PI positivity of WT MEFs treated with BH3 mimetic (30 μM ABT-199, 10 μM A-1331852, 10 μM S63845, 10 μM ABT-737, 500 nM Obatoclax, or vehicle (Veh) (DMSO) in the presence of cell death inducers 5 µM ionomycin (Iono) for 20 h, 5 nM staurosporine (St) for 12 h, or untreated (Unt). Three independent experiments (*n* = 3) were performed for every panel or otherwise noted. *p ≤ 0.05 for comparison to the Unt control for all BH3 mimetic treatments. ^#^ *p* ≤ 0.05 for comparison of the BH3 mimetic treatment samples to the corresponding stress (St or Iono) in the Veh group.

To determine if the inhibition of various anti-apoptotic Bcl-2 family members through the treatment of BH3 mimetics can exacerbate necrotic cell death, we first determined the amount of each anti-apoptotic Bcl-2 family member expressed at baseline within the mitochondria in WT MEFs by using tandem mass tag-mass spectrometry (TMT-MS) (Figure 1A). The expression of each anti-apoptotic Bcl-2 family member detected by TMT-MS was confirmed by western blot analysis (Figure 1B). Bcl-xL and Mcl-1 were found to be the most abundant anti-apoptotic Bcl-2 family members followed by Bcl-2 in WT MEF mitochondria. WT MEFs were treated with low concentrations of BH3 mimetics (mimetics ABT-199 (Bcl-2 inhibitor), A-1331852 (Bcl-xL inhibitor), S63845 (Mcl-1 inhibitor), ABT-737 (Bcl-2 and Bcl-xL inhibitor), or Obatoclax (Bcl-2, Bcl-xL, and Mcl-1 inhibitor) in the presence or absence of low dose of ionomycin (iono) (5 µM for 20 h) or staurosporine (St) (5 nM for 12 h). Alone, each treatment led to a small increase in cell death, however, the BH3 mimetics exacerbated both apoptosis induced by staurosporine and necrosis induced by ionomycin in WT MEFs (Figure 1C). These data suggest that the anti-apoptotic Bcl-2 family members play a protective role against Ca^2+^ overload-induced cell death, which is indicative of these members playing a protective role against MPTP-dependent necrosis.

### BH3 Mimetics Reduce Calcium Retention Capacity of Mitochondria by Sensitizing MPTP Opening

**FIGURE 2:**
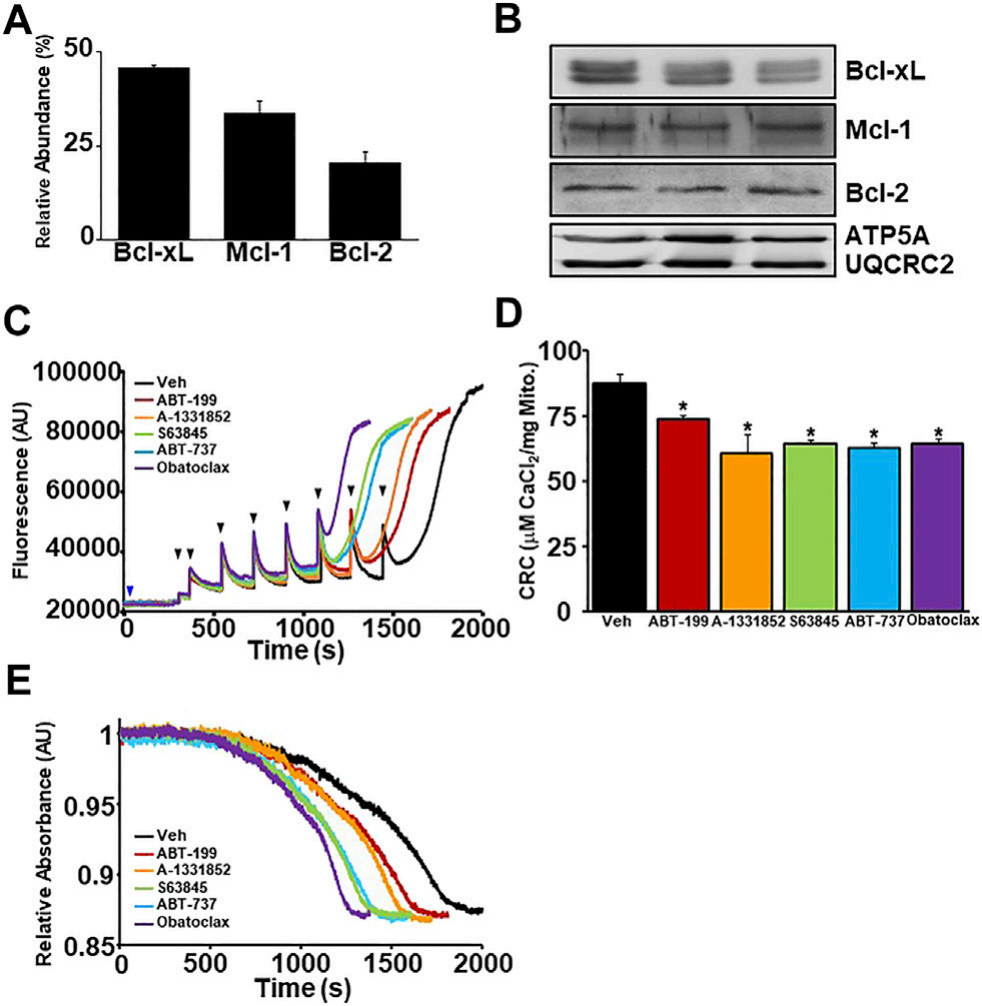
BH3 mimetics decrease mitochondrial CRC and sensitize MPTP opening. **(A)** Quantitation of the relative abundance of anti-apoptotic Bcl-2 family members (Bcl-2, Bcl-xL, and Mcl-1) from enriched liver mitochondrial fractions isolated from mouse livers measured by tandem-mass-tagging mass spectrometry (*n* = 5). **(B)** Western blots for anti-apoptotic Bcl-2 (Bcl-2, Bcl-xL, and Mcl-1) and Ox. Phos. (ATP5A and UQCRC2) (loading control) using enriched mitochondrial lysates isolated from mouse livers. **(C)** Representative traces of mitochondrial calcium uptake of WT liver mitochondria treated with either 200 nM ABT-199 (red), 100 nM A-1331852 (orange), 10 nM S63845 (green), 100 nM ABT-737 (blue), 10 nM Obatoclax (purple), or with the vehicle control (black). The blue arrow represents the addition of the BH3 mimetics. The black arrowheads represent the addition of 20 μM CaCl_2._ **(D)** Quantification of mitochondrial calcium retention capacity (CRC) calculated from mitochondrial calcium uptake traces, as in **(C)**. **(E)** Representative trace of the corresponding mitochondrial swelling to **(C)** of WT liver mitochondria treated with BH3 mimetics and CaCl_2_. Three independent experiments (*n* = 3) were performed for every panel or otherwise noted.

To investigate the effects of BH3 mimetics on the MPTP, we utilized *ex vivo* liver mitochondrial isolations due to their accessibility and abundance. Prior to examining the effect of the mimetics on MPTP kinetics, we performed TMT-MS on isolated liver mitochondrial fractions to elucidate the abundance of the anti-apoptotic Bcl-2 family members in liver mitochondria at baseline (Figure 2A). Bcl-xL is the most abundantly expressed anti-apoptotic Bcl-2 protein, followed by Mcl-1 and then Bcl-2. The expression of each anti-apoptotic member detected by TMT-MS was confirmed by western blot analysis (Figure 2B). To determine whether BH3 mimetics affect mitochondrial Ca^2+^ uptake and/or retention and MPTP sensitivity, WT liver mitochondria were incubated with increasing concentrations of each BH3 mimetic (0.5, 1, 10, 50, 100 nM, 1 µM) prior to being challenged with Ca^2+^ boluses. Following incubation, mitochondrial Ca^2+^ retention capacity (CRC) and swelling were analysed (Supplementary Figures S1A–K). All BH3 mimetics tested were able to sensitize MPTP opening in a dose-dependent manner. However, the multi-targeted BH3 mimetics sensitize the MPTP greater than the single targeted mimetics (Supplementary Figure S1K). Ultimately, we decided to utilize the lowest possible dose of each mimetic that was able to decrease CRC and sensitize MPTP opening to ensure target specificity. These chosen concentrations of each BH3 mimetic are represented in Figures 2C–E.

### MPTP Sensitization by the BH3 Mimetics Persists in CypD Deletion/Inhibition or ANT Antagonized Mitochondria

**FIGURE 3:**
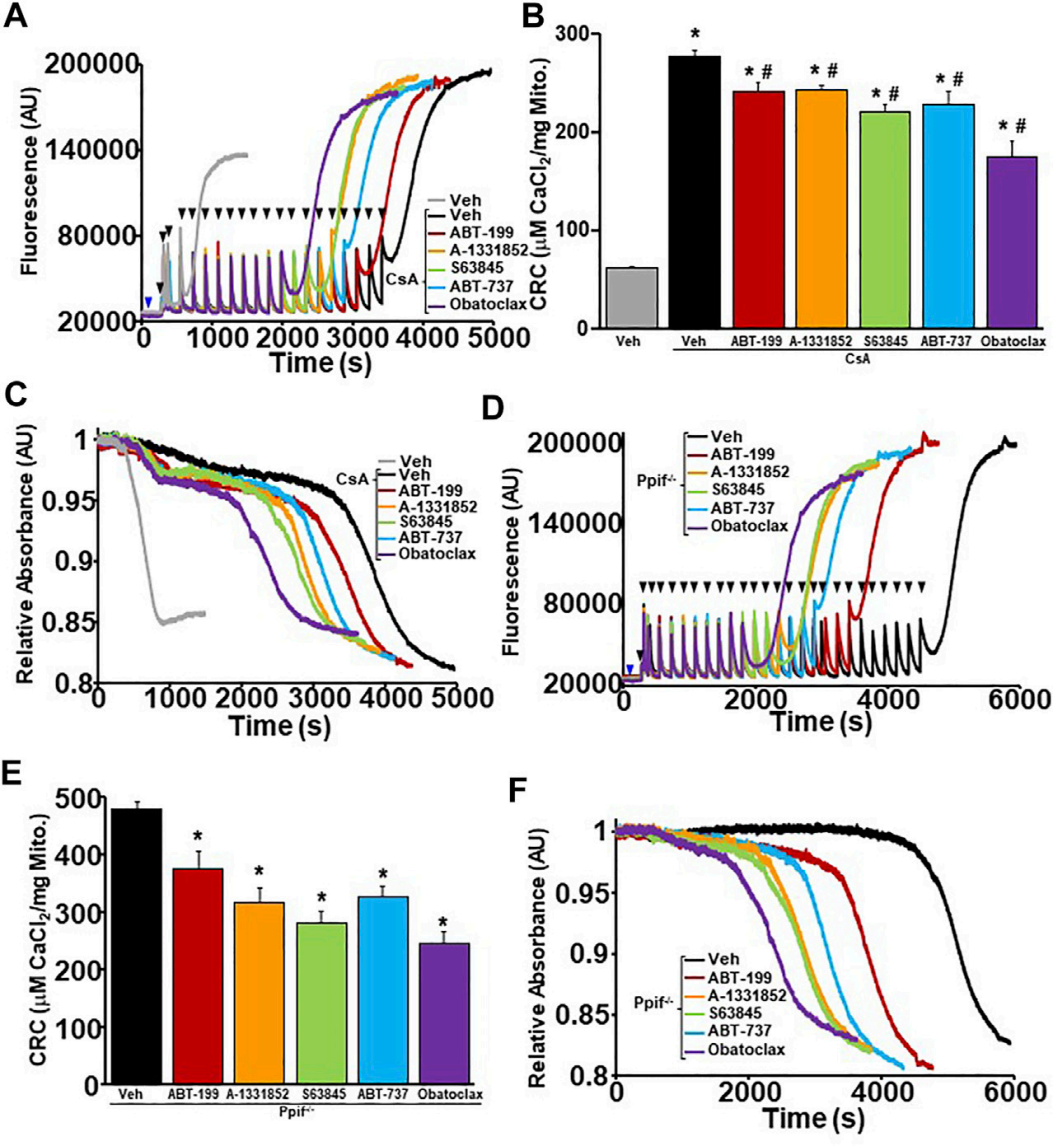
CypD inhibited or null liver mitochondria have reduced mitochondrial CRC and sensitized MPTP opening upon treatment of BH3 mimetics. **(A)** Representative traces of mitochondrial calcium uptake of WT liver mitochondria pre-treated with 2 µM cyclosporine A (CsA) and then with either 200 nM ABT-199 (red), 100 nM A-1331852 (orange), 10 nM S63845 (green), 100 nM ABT-737 (blue), 10 nM Obatoclax (purple), or with the vehicle control (black). Control WT liver mitochondria without CsA pretreatment is shown in grey. The blue arrow represents the addition of the BH3 mimetics. The black arrowheads represent the addition of 40 μM CaCl_2._ **(B)** Quantification of mitochondrial calcium retention capacity (CRC) calculated from mitochondrial calcium uptake traces, as in **(A)**. **(C)** Representative trace of corresponding mitochondrial swelling to **(A)** of WT liver mitochondria pre-treated with CsA and then treated with BH3 mimetics and CaCl_2_. **(D)** Representative traces of mitochondrial calcium uptake of *ppif* ^*−/−*^ liver mitochondria treated with either 200 nM ABT-199 (red), 100 nM A-1331852 (orange), 10 nM S63845 (green), 100 nM ABT-737 (blue), 10 nM Obatoclax (purple), or with the vehicle control (black). The blue arrow represents the addition of the BH3 mimetics. The black arrowheads represent the addition of 40 μM CaCl_2._ **(E)** Quantification of mitochondrial calcium retention capacity (CRC) calculated from mitochondrial calcium uptake traces, as in **(D)**. **(F)** Representative trace of the corresponding mitochondrial swelling to **(D)** of *ppif* ^*−/−*^ liver mitochondria treated with BH3 mimetics and CaCl_2_. Three independent experiments (*n* = 3) were performed for every panel. *p ≤ 0.05 for comparison the Veh. ^#^ *p* ≤ 0.05 for comparison the Veh w/CsA.

CypD and the ANT family are two major regulators of the MPTP, whereas CypD triggers pore opening from the matrix of mitochondria via its isomerase activity, while the ANT family can function as a pore-forming component of the MPTP itself (Brustovetsky and Klingenberg, 1996; Rück et al., 1998; Brustovetsky et al., 2002; Karch and Molkentin, 2014). When either regulator is deleted or inhibited, the MPTP becomes desensitized to Ca^2+^-induced opening. However, when both regulators are inhibited against their MPTP functions, Ca^2+^-induced MPTP opening is completely inhibited (Karch et al., 2019). Here, we wanted to probe how the inhibition of the anti-apoptotic Bcl-2 family members may lead to MPTP sensitization by determining how they affect the MPTP when the critical regulators are deleted or inhibited. CRC/swelling assays were performed on WT liver mitochondria pre-treated with with cyclosporine A (CsA), an inhibitor of CypD, and BH3 mimetics to determine whether anti-apoptotic Bcl-2 proteins sensitize the MPTP independently of CypD. Mitochondrial CRC was significantly decreased and MPTP opening occurred with less Ca^2+^ when the BH3 mimetics were present when CypD was inhibited by CsA (Figures 3A–C). We genetically confirmed these results, using liver mitochondria isolated from *Ppif* ^*−/−*^ mice (*Ppif* being the gene that encodes CypD) and treating these mitochondria with the various BH3 mimetics. Similar to pharmacological inhibition, the CypD null mitochondria had reduced CRC due to sensitization of the MPTP in the presence of all BH3 mimetics (Figures 3D–F). Notably, there was a correlative trend for the multi-targeted BH3 mimetics (ABT-737 and Obatoclax) to have a greater effect than the single targeted mimetics.

**FIGURE 4:**
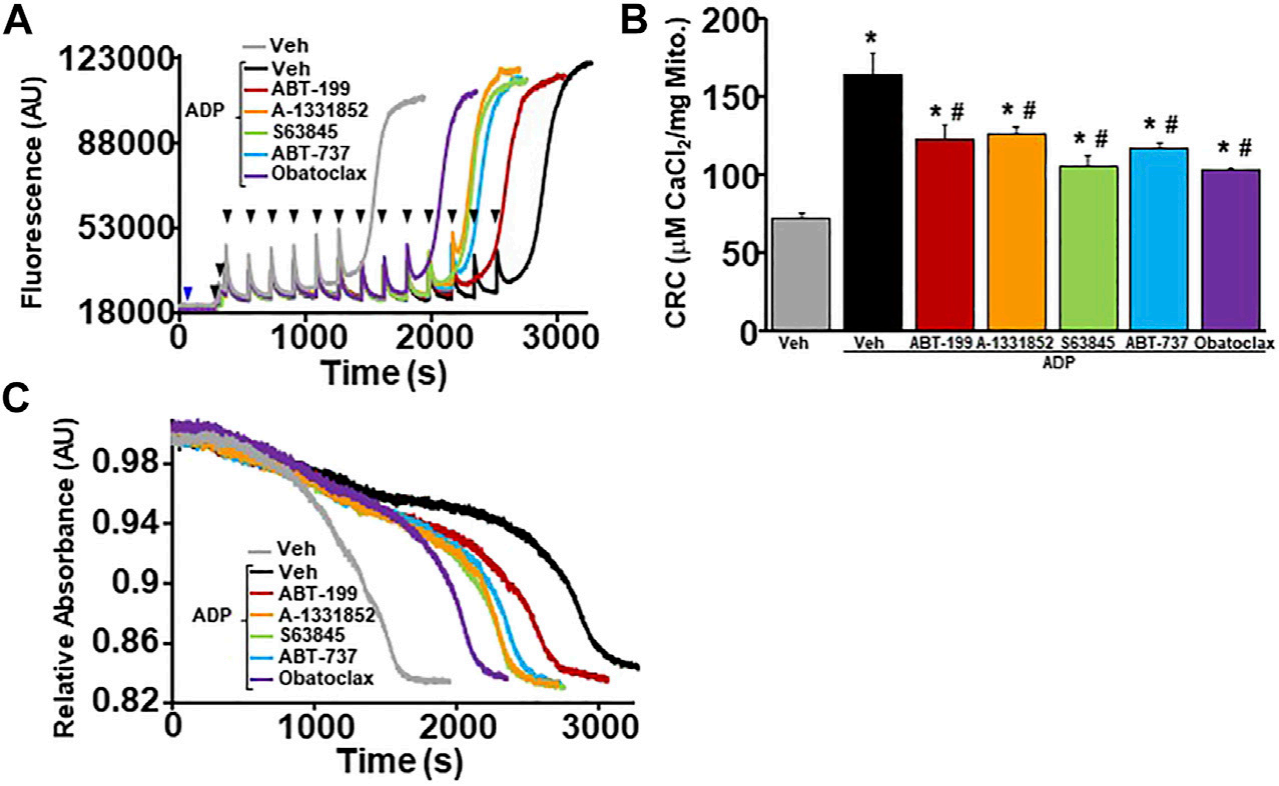
BH3 mimetics reduced mitochondrial CRC and sensitize MPTP opening in the presence of ADP. **(A)** Representative traces of mitochondrial calcium uptake of WT liver mitochondria pre-treated with 300 µM adenosine diphosphate (ADP) and then with either 200 nM ABT-199 (red), 100 nM A-1331852 (orange), 10 nM S63845 (green), 100 nM ABT-737 (blue), 10 nM Obatoclax (purple), or with the vehicle control (black). Control WT liver mitochondria without ADP pretreatment is shown in grey. The blue arrow represents the addition of the BH3 mimetics. The black arrowheads represent the addition of 20 μM CaCl_2._ **(B)** Quantification of mitochondrial calcium retention capacity (CRC) calculated from mitochondrial calcium uptake traces, as in **(A)**. **(C)** Representative trace of corresponding mitochondrial swelling to **(A)** of WT liver mitochondria pre-treated with ADP and then treated with BH3 mimetics and CaCl_2_. Three independent experiments (*n* = 3) were performed for every panel. *p ≤ 0.05 for comparison the Veh. ^#^ *p* ≤ 0.05 for comparison the Veh w/ADP.

**FIGURE 5:**
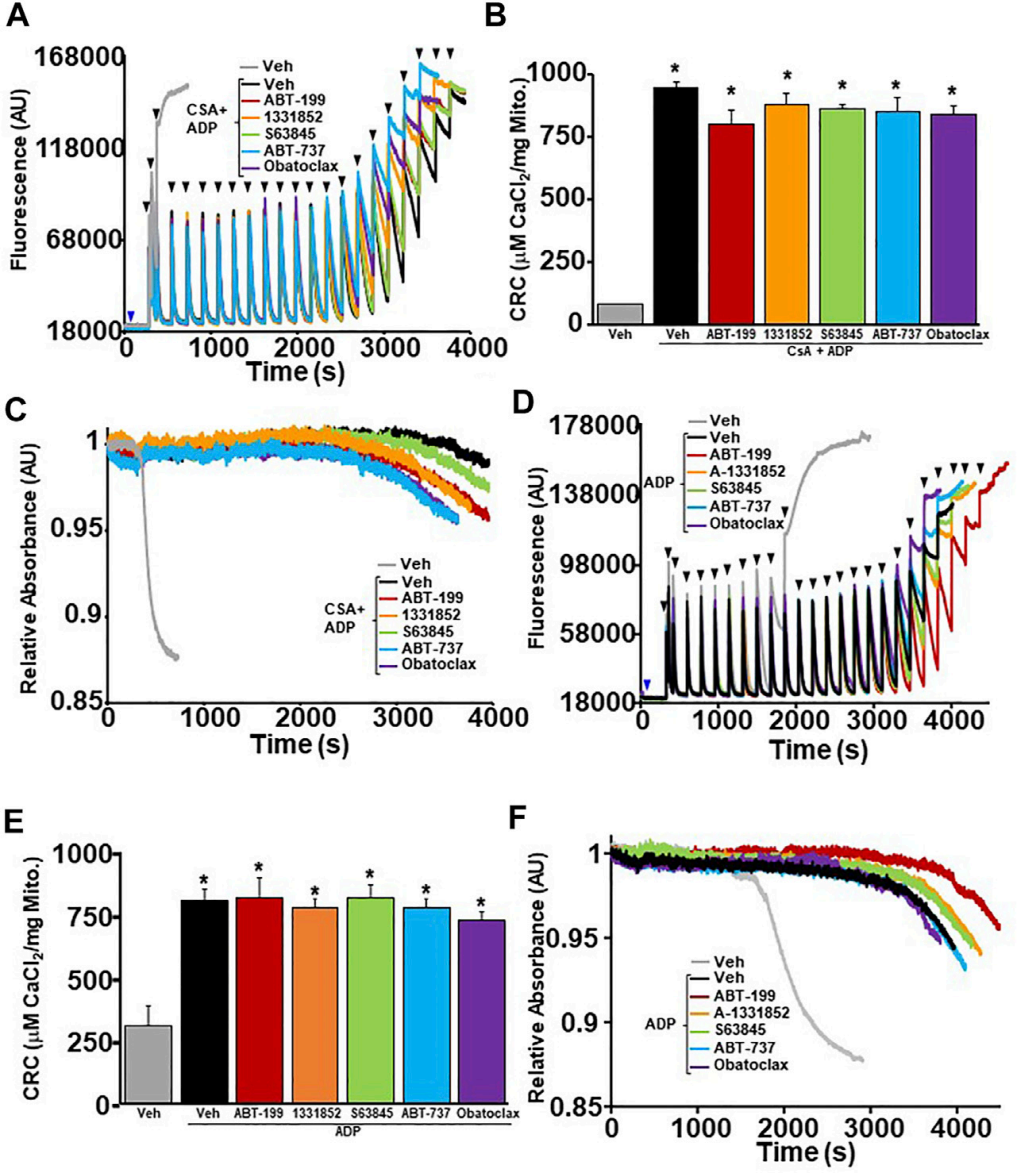
MPTP inhibition by dual treatment of CsA and ADP prevents BH3 mimetics ability to effect mitochondrial CRC. **(A)** Representative traces of mitochondrial calcium uptake of WT liver mitochondria pre-treated with 300 µM adenosine diphosphate (ADP) and 2 µM cyclosporine A (CsA). Following a 5 min incubation, they were then treated with either 200 nM ABT-199 (red), 100 nM A-1331852 (orange), 10 nM S63845 (green), 100 nM ABT-737 (blue), 10 nM Obatoclax (purple), or with the vehicle control (black). Control WT liver mitochondria without ADP + CsA pretreatment is shown in grey. The blue arrow represents the addition of the vehicle or BH3 mimetics. The black arrowheads represent the addition of 80 μM CaCl_2._ **(B)** Quantification of mitochondrial calcium retention capacity (CRC) was calculated from mitochondrial calcium uptake traces, as in **(A)**. **(C)** Corresponding mitochondrial swelling representative trace to **(A)** of WT liver mitochondria pre-treated with ADP and then treated with BH3 mimetics and CaCl_2_. Three independent experiments (*n* = 3) were performed for every panel. *p ≤ 0.05 for comparison of the Veh without CsA and ADP treatment. **(D)** Representative traces of mitochondrial calcium uptake of CypD null liver mitochondria pre-treated with 300 µM ADP. Following a 5 min incubation, the mitochondira were then treated with either 200 nM ABT-199 (red), 100 nM A-1331852 (orange), 10 nM S63845 (green), 100 nM ABT-737 (blue), 10 nM Obatoclax (purple, or with vehicle control (black). Control CypD null liver mitochondria without ADP pretreatment is shown in grey. The blue arrow represents the addition of the BH3 mimetics or vehicle. The black arrowheads represent the addition of 80 µM CaCl_2_. **(E)** Quantification of mitochondrial CRC was calculated from mitochondrial calcium uptake traces, as in **(D)**. **(F)** Corresponding mitochondrial swelling representative trace to **(D)** of *ppif* ^*−/−*^ liver mitochondria pre-treated with ADP and then treated with BH3 mimetics and CaCl_2_. Three independent experiments (*n* = 3) were performed for every panel. *p* ≤ 0.05 for comparison of the Veh without ADP treatment.

In order to determine whether BH3 mimetics sensitizes MPTP opening through the ANT family, WT liver mitochondria were isolated and pre-treated with ADP followed by BH3 mimetics treatment before performing CRC analysis. ADP is a substrate of the ANT family that has an inhibitory effect on the ability of the ANT family to contribute to MPTP opening by stabilizing the location of their nucleotide binding in the matrix (“m”-state) (Buchanan et al., 1976; Haworth and Hunter, 2000). The amount of Ca^2+^ required for MPTP opening decreased significantly in WT liver mitochondria pre-treated with ADP in the presence of each BH3 mimetics (Figures 4A–C). Again, BH3 mimetics targeting multiple anti-apoptotic Bcl-2 family members induced mitochondrial swelling prior to single targeting BH3 mimetics. To determine if the mimetics were able to sensitize the MPTP when both the ANTs and CypD were antagonized, we treated WT liver mitochondria with a combination of CsA and ADP, followed by BH3 mimetics before challenging them with Ca^2+^ boluses and measuring mitochondrial CRC/swelling. As we have previously reported, this combination treatment impressively increases CRC to the point of Ca^2+^ saturation with the absence of a large magnitude-swelling event (Figures 5A,C). Under this MPTP-inhibited state, treatment with all the various BH3 mimetics was unable to reduce CRC and restore mitochondrial swelling (Figures 5A–C). To confirm these results genetically, we isolated mitochondria from *Ppif* null mice and treated them with ADP to inhibit the MPTP. Similarly, to the combined treatment of CsA and ADP, ADP treated CypD null mitochondria do not engage MPTP opening and MPTP activity is not sensitized or restored in the presence of any of the BH3 mimetics (Figures 5D–F). Thus, BH3 mimetic-dependent sensitization of the MPTP cannot supersede inhibition of the MPTP by dual treatment of ADP and CsA or CypD deletion with ADP.

### BH3 Mimetic Sensitization of the MPTP and Exacerbation of Necrotic Cell Death is Dependent on the Expression of Bax and Bak

**FIGURE 6:**
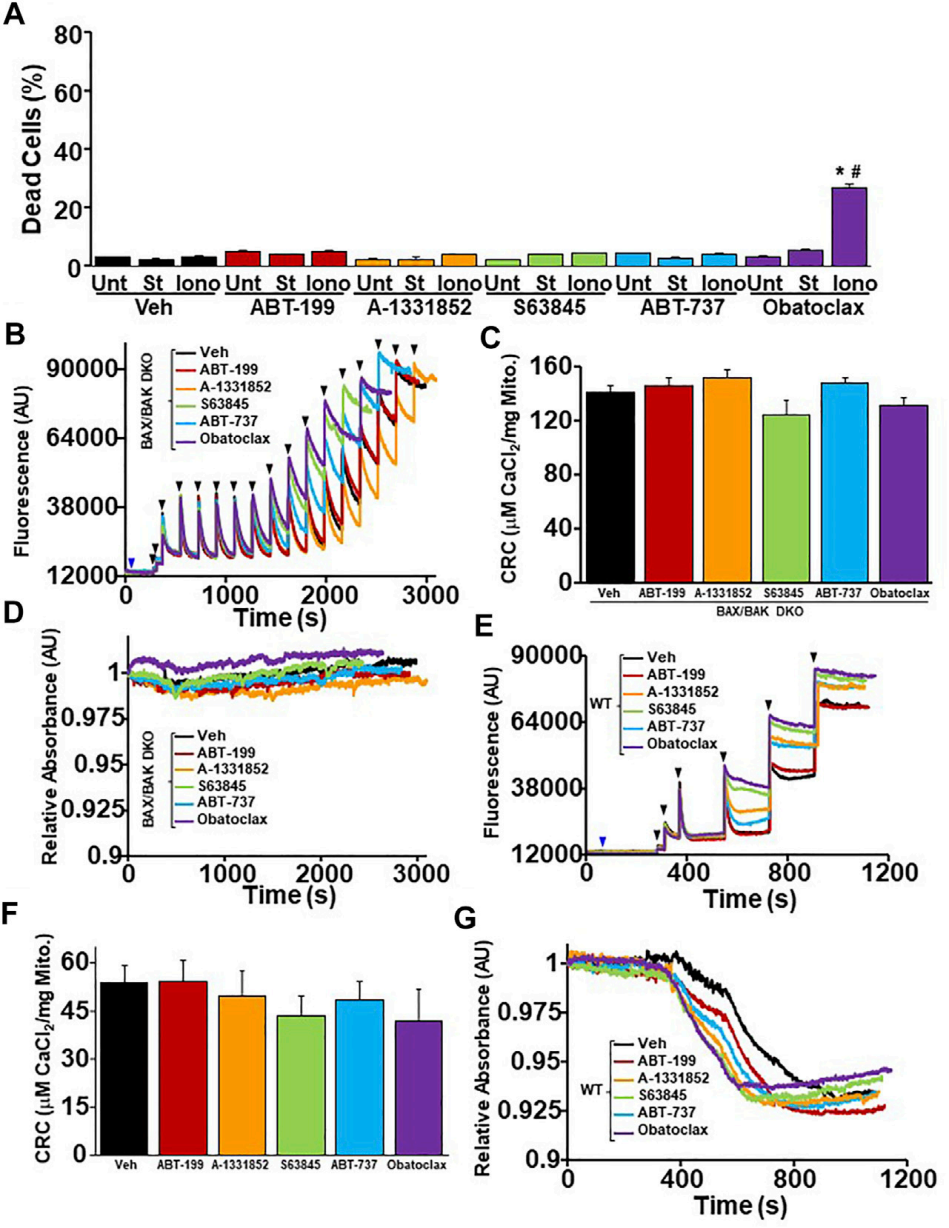
The ability of BH3 mimetics to reduced mitochondrial CRC and sensitize MPTP opening is dependent on Bax/Bak. **(A)** Quantitation of cell death by propidium iodide inclusion quantified using flow cytometry. BAX/BAK double knockout (DKO) MEFs were treated with the various BH3 mimetic (30 μM ABT-199, 10 μM A-1331852, 10 μM S63845, 10 μM ABT-737, 500 nM Obatoclax) and 5 µM ionomycin (Iono) for 20 h or 5 nM staurosporine (St) for 12 hr. **(B)** Representative traces of mitochondrial calcium uptake of DKO MEF mitochondria treated with either 200 nM ABT-199 (red), 100 nM A-1331852 (orange), 10 nM S63845 (green), 100 nM ABT-737 (blue), 10 nM Obatoclax (purple), or with the vehicle (Veh) control (black). The blue arrow represents the addition of the BH3 mimetics. The black arrowheads represent the addition of 20 μM CaCl_2_. **(C)** Quantification of mitochondrial calcium retention capacity (CRC) calculated from mitochondrial calcium uptake traces, as in **(B)**. **(D)** Representative trace of the corresponding mitochondrial swelling to **(C)** of DKO MEF mitochondria treated with BH3 mimetics and CaCl_2_. **(E)** Representative traces of mitochondrial calcium uptake of WT MEF mitochondria treated with either 200 nM ABT-199 (red), 100 nM A-1331852 (orange), 10 nM S63845 (green), 100 nM ABT-737 (blue), 10 nM Obatoclax (purple), or with the vehicle (Veh) control (black). The blue arrow represents the addition of the BH3 mimetics. The black arrowheads represent the addition of 20 μM CaCl_2_. **(F)** Quantification of mitochondrial calcium retention capacity (CRC) calculated from mitochondrial calcium uptake traces, as in **(E)**. **(G)** Representative trace of the corresponding mitochondrial swelling to (E) of WT MEF mitochondria treated with BH3 mimetics and CaCl_2_. Three independent experiments (*n* = 3) were performed for every panel. *p ≤ 0.05 for comparison to the Unt control within the same BH3 mimetic treatment. ^#^ *p* ≤ 0.05 for comparison of the BH3 mimetic treatment samples to the corresponding stress (St or Iono) in the Veh group.

The inhibition of anti-apoptotic Bcl-2 family members by BH3 mimetics pushes the Bcl-2 family towards the activation and oligomerization of Bax and Bak to sensitize cells to apoptotic cell death (Villalobos-Ortiz et al., 2020). To determine if the exacerbation of necrotic cell death by BH3 mimetic treatment in WT MEFs is dependent on the expression of Bax and Bak, we treated *Bax/Bak1* DKO MEFs with BH3 mimetics in the presence of staurosporine (5 nM) or ionomycin (5 µM) before measuring cell death using PI staining and flow cytometry. *Bax/Bak1* DKO MEFs were resistant to BH3 mimetic exacerbation of both apoptotic and necrotic cell death compared to WT MEFs, except for Obatoclax, which induced a low level of toxicity when combined with ionomycin (Figures 1A, 6A). Mitochondria isolated from WT and *Bax/Bak1* DKO MEFs treated with BH3 mimetics prior to Ca^2+^ boluses demonstrated that BH3 mimetics sensitize MPTP opening in WT mitochondria, while Bax/Bak null mitochondria were refractory to the treatment of mimetics and Ca^2+^ (Figures 6B–G). Thus, the inhibition of the anti-apoptotic Bcl-2 family members function through Bax and Bak to sensitize mitochondria to Ca^2+^-dependent MPTP opening and necrotic cell death. Surprisingly, the treatment of ADP and/or CsA on Bax/Bak null mitochondria did not enhance mitochondrial CRC or alter the kinetics of mitochondrial swelling compare to WT control mitochondria (Supplementary Figures 2A–F). These data confirm that Bax and Bak are critical outer membrane mitochondrial regulators of the MPTP, as previously suggested (Karch et al., 2013).

## Discussion

The proapoptotic pore-forming Bcl-2 family members, Bax and Bak, have been implicated in both apoptotic cell death and MPTP-dependent necrotic cell death (Karch et al., 2013). During apoptosis, Bax and Bak form homo/hetero oligomers, which lead to a large increase in outer mitochondrial permeability (Westphal et al., 2014). While during MPTP-dependent necrosis non-oligomeric Bax and Bak are sufficient for MPTP-dependent mitochondrial dysfunction and subsequent necrotic cell death (Karch et al., 2013). Recently, reports suggest that over activation of Bax leads to inner membrane rupture and mitochondrial DNA release, which may act like a damage-associated molecular pattern to initiate necrotic cell death (McArthur et al., 2018; Riley et al., 2018). Upstream of Bax and Bak are the other Bcl-2 family members that work in balance to regulate the activity of Bax and Bak (Youle and Strasser, 2008). Here, we utilized selective inhibitors targeting the different anti-apoptotic Bcl-2 family members expressed at the level of the mitochondria to determine if they influence mitochondrial matrix Ca^2+^ retention capacity and MPTP sensitivity. Bcl-2, Bcl-xL, and Mcl-1 have been previously reported to play an inhibitory against MPTP opening, however, the mechanism by which each family member plays to elicit this negative regulation is unknown. One potential way is through their ability to regulate voltage dependent anion channels (VDACs), which were once suggested to be the outer membrane component of the MPTP (Tsujimoto and Shimizu, 2000; Karch and Molkentin, 2014). Another, through their ability to bind to the F_1_F_o_ ATP-synthase, which has been suggested to be one of the inner membrane pore-forming components of the MPTP (Alavian et al., 2011; Bonora et al., 2013; Giorgio et al., 2013; Park et al., 2017). In addition, Mcl-1 can localize to the outer membrane, the inner membrane, as well as the matrix of the mitochondria, which may enable Mcl-1 to effect MPTP sensitivity in unique ways compared to other members that only reside on the outer membrane (Perciavalle et al., 2012). Finally, all anti-apoptotic Bcl-2 family members regulate the activity of Bax and Bak, which have also been suggested to be the outer membrane component of the MPTP (Karch et al., 2013).

Our data suggests that the inhibition of each individual anti-apoptotic Bcl-2 family member leads to decreased mitochondrial CRC due to sensitization of MPTP opening. When the MPTP was desensitized by treatment with ADP, CsA, or by genetic removal of Ppif, inhibition of each individual anti-apoptotic Bcl-2 family member still resulted in decreased mitochondrial CRC due to sensitization of MPTP opening. However, when the MPTP was fully inhibited by the combination treatment of CsA and ADP, inhibition of each Bcl-2 family member no longer effected CRC and was unable to sensitize or restore MPTP opening. Since the inhibition of all anti apoptotic Bcl-2 family members had the similar ability to sensitize the MPTP, these data suggest that all these family members function to sensitize mitochondria to MPTP opening through a common mechanism. This mechanism is dependent on the expression of Bax and Bak, since the inhibition of each individual and multiple anti-apoptotic Bcl-2 family members do not reduce mitochondrial CRC or restore mitochondrial swelling in Bax/Bak null mitochondria. Together, these data confirm that outer membrane regulation of the MPTP is through Bax and Bak and suggest that the activation/oligomerization of Bax/Bak on the outer mitochondrial membrane somehow regulates matrix Ca^2+^ levels by sensitizing the MPTP. The mechanistic interplay between mitochondrial outer membrane permeabilization and matrix Ca^2+^ sensitivity requires further investigation.

In regard to cell death, our data continues to blur the lines between apoptosis and necrosis. The Bcl-2 family regulates outer mitochondrial membrane permeabilization through Bax and Bak, which we show can influence inner mitochondrial membrane permeabilization by sensitizing of the MPTP to Ca^2+^. This capability of the Bcl-2 family links apoptotic cell death to necrotic cell death pathways that are dependent on mitochondrial dysfunction. The point of distinction between apoptosis and necrosis depends on the level of matrix Ca^2+^ and the oligomerization status of Bax and Bak. Stressors that influence Bax/Bak oligomerization in the absence of a Ca^2+^ insult will likely lead to caspase-dependent apoptotic cell death, whereas stressors that lead to a large amount of mitochondrial Ca^2+^/ROS in the absence of Bax/Bak oligomerization will likely lead to MPTP-dependent necrosis. Stressors that simultaneously induce Bax/Bak oligomerization and increase mitochondrial Ca^2+^ may require less Ca^2+^ to induce mitochondrial dysfunction due to increased outer mitochondrial membrane permeability through Bax and Bak.

## Conclusion

We have found that the MPTP sensitivity to matrix Ca^2+^ is regulated by BH3 mimetic inhibition of the anti-apoptotic Bcl-2 family members localized to mitochondria. Individual or compound BH3 mimetic (ABT-199, A-1331852, ABT-737, S63845, and Obatoclax) inhibition of Bcl-2, Bcl-xL, or Mcl-1 leads to reduced mitochondrial CRC due to sensitization of the MPTP. This sensitization persists in mitochondria that are desensitized to undergo MPTP opening through CypD inhibition or by ADP treatment. However, when the MPTP inhibition is achieved by the combined treatment of CsA and ADP or deletion of Ppif and ADP treatment, BH3 mimetics no longer effect the MPTP or CRC. In addition, the inhibition of the anti-apoptotic Bcl-2 family’s effect on MPTP sensitivity and necrotic cell death is dependent on the expression of Bax and Bak. Indeed, in the absence of Bax and Bak, all of the BH3 mimetics used in this study do not reduce mitochondrial CRC or effect mitochondrial swelling. Our data highlights the interplay between outer membrane permeabilization through the Bcl-2 family and MPTP sensitivity to matrix Ca^2+^. Not only is the expression of Bax and Bak required for MPTP-dependent mitochondrial dysfunction to occur, but also increased oligomerization of Bax and Bak reduces the amount of Ca^2+^ needed to engage MPTP opening and subsequent necrotic cell death. Further mechanistic understanding of how outer mitochondrial membrane permeability by the Bcl-2 family influences mitochondrial matrix CRC is required in order to design therapeutic strategies to prevent pathological cell death that involves MPTP-dependent mitochondrial dysfunction.

## Data Availability

The raw data supporting the conclusion of this article will be made available by the authors, without undue reservation.

## Supplementary Material

Supplementary Figure 1 Dose dependent reduction in mitochondrial CRC and MPTP sensitization by various BH3 mimetics. **(A)** Representative traces of mitochondrial calcium uptake of WT liver mitochondria treated with ABT-199 (1 nM, 10 nM, 50 nM, 100 nM, and 1 µM). The blue arrowhead represents the addition of the BH3 mimetics. The black arrowheads represent the addition of 20 μM CaCl2. **(B)** Representative trace of the corresponding mitochondrial swelling to **(A)**. **(C)** Representative traces of mitochondrial calcium uptake of WT liver mitochondria treated with A-1131852 (1 nM, 10 nM, 50 nM, 100 nM, and 1 µM). The blue arrowhead represents the addition of the BH3 mimetics. The black arrowheads represent the addition of 20 μM CaCl2. **(D)** Representative trace of the corresponding mitochondrial swelling to **(C)**. **(E)** Representative traces of mitochondrial calcium uptake of WT liver mitochondria treated with S63845 (1 nM, 10 nM, 50 nM, 100 nM, and 1 µM). The blue arrowhead represents the addition of the BH3 mimetics. The black arrowheads represent the addition of 20 μM CaCl2. **(F)** Representative trace of the corresponding mitochondrial swelling to **(E)**. **(G)** Representative traces of mitochondrial calcium uptake of WT liver mitochondria treated with ABT-737 (1 nM, 10 nM, 50 nM, 100 nM, and 1 µM). The blue arrowhead represents the addition of the BH3 mimetics. The black arrowheads represent the addition of 20 μM CaCl2. **(H)** Representative trace of the corresponding mitochondrial swelling to **(G)**. **(I)** Representative traces of mitochondrial calcium uptake of WT liver mitochondria treated with Obatoclax (0.5 nM, 1 nM, 10 nM, 50 nM, and 100 nM). The blue arrowhead represents the addition of the BH3 mimetics. The black arrowheads represent the addition of 20 μM CaCl2. **(J)** Representative trace of the corresponding mitochondrial swelling to **(I)**. **(K)** Quantification of mitochondrial calcium retention capacity (CRC) calculated from mitochondrial calcium uptake traces, as in **(A,C,E,G,I)**. Three independent experiments (*n*= 3) were performed for every panel.

Supplementary Figure 2 CsA and ADP do not increase mitochondrial CRC or desensitize MPTP opening in BAK/BAK null MEF mitochondria. **(A)** Representative traces of mitochondrial calcium uptake of WT MEF mitochondria treated with either DMSO (veh) (black), 300 µM ADP, 2 µM CsA, or a combination of both ADP and CsA. The black arrowheads represent the addition of 20 μM CaCl2. **(B)** Quantification of mitochondrial calcium retention capacity (CRC) calculated from mitochondrial calcium uptake traces, as in **(A)**. **(C)** Representative trace of the corresponding mitochondrial swelling to **(A)** of WT MEF mitochondria treated with MPTP inhibitors and CaCl2. **(D)** Representative traces of mitochondrial calcium uptake of DKO MEF mitochondria treated with either DMSO (veh) (black), 300 µM ADP, 2 µM CsA, or a combination of both ADP and CsA. The black arrowheads represent the addition of 20 μM CaCl2. **(E)** Quantification of mitochondrial calcium retention capacity (CRC) calculated from mitochondrial calcium uptake traces, as in **(D)**. **(F)** Representative trace of the corresponding mitochondrial swelling to **(D)** of DKO MEF mitochondria treated with MPTP inhibitors and CaCl2. Three independent experiments (*n*= 3) were performed for every panel. **p* ≤ 0.05 for comparison the Veh.
