# Supplementary figures and images for: Inhibition of the Anti-Apoptotic Bcl-2 Family by BH3 Mimetics Sensitize the Mitochondrial Permeability Transition Pore Through Bax and Bak
Source: Front Cell Dev Biol. 2021 Dec 1;9:765973. doi: 10.3389/fcell.2021.765973 (PMC8672142; doi:10.3389/fcell.2021.765973)

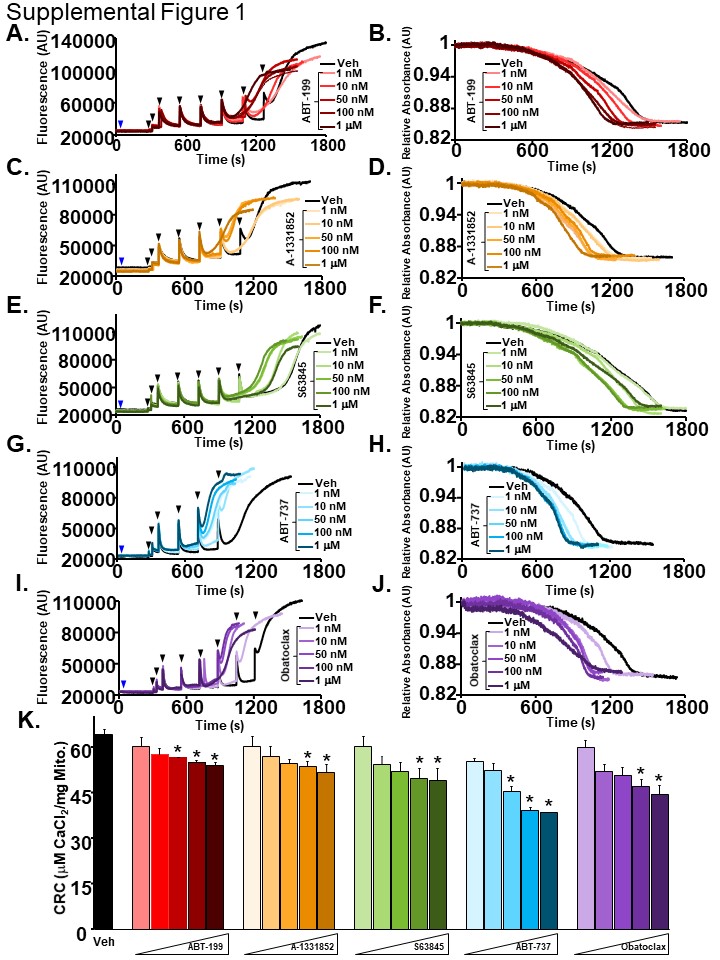

Supplement: Supplementary file 1 [file Image1.jpeg]

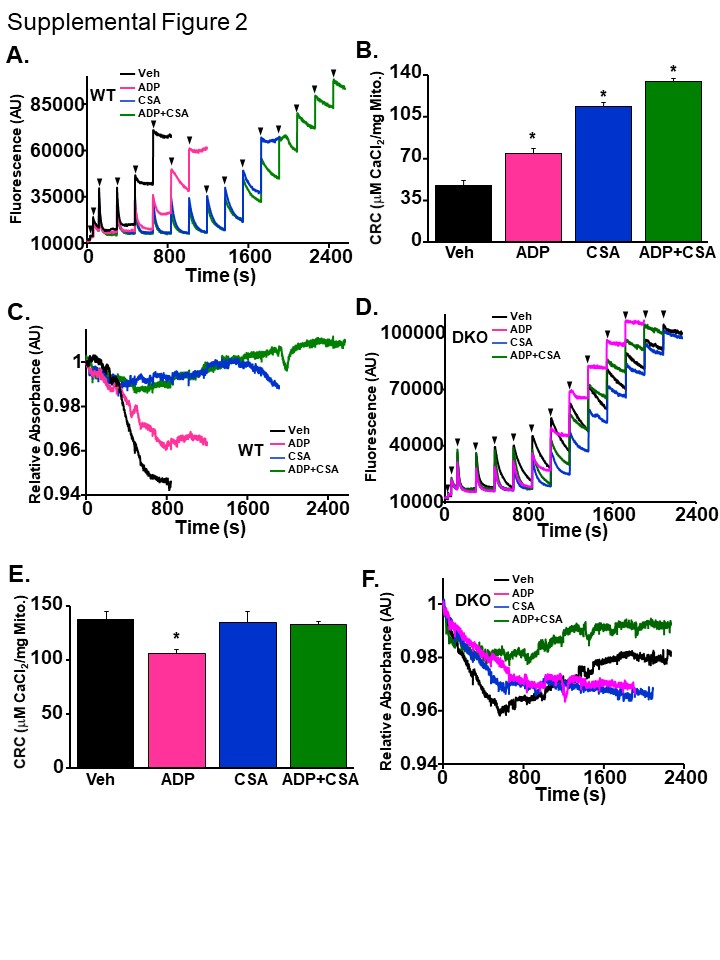

Supplement: Supplementary file 2 [file Image2.jpeg]
